# Supplementary material for: Linsitinib inhibits IGF-1-induced cell proliferation and hyaluronic acid secretion by suppressing PI3K/Akt and ERK pathway in orbital fibroblasts from patients with thyroid-associated ophthalmopathy
Source: PLoS One. 2024 Dec 18;19(12):e0311093. doi: 10.1371/journal.pone.0311093 (PMC11654993; doi:10.1371/journal.pone.0311093)
Supplement: S1 Fig — Representative histogram of the gated cells in the G0/G1, S, and G2/M phases of linsitinib-pretreated or/and IGF-1-treated OFs from TAO patients. OFs from TAO patients were plated at a concentration of 2 x 10^5^ cells/well in a 6-well plate. After 24 h, the cells were pre-treated with linsitinib at the indicated concentrations for 2 h, followed by treatment with 50 ng/ml IGF-1 for 24 h. After 24 h, cells were collected and stained with propidium iodide (PI) and their DNA contents were analyzed by flow cytometry. (PDF) [file pone.0311093.s001.pdf]

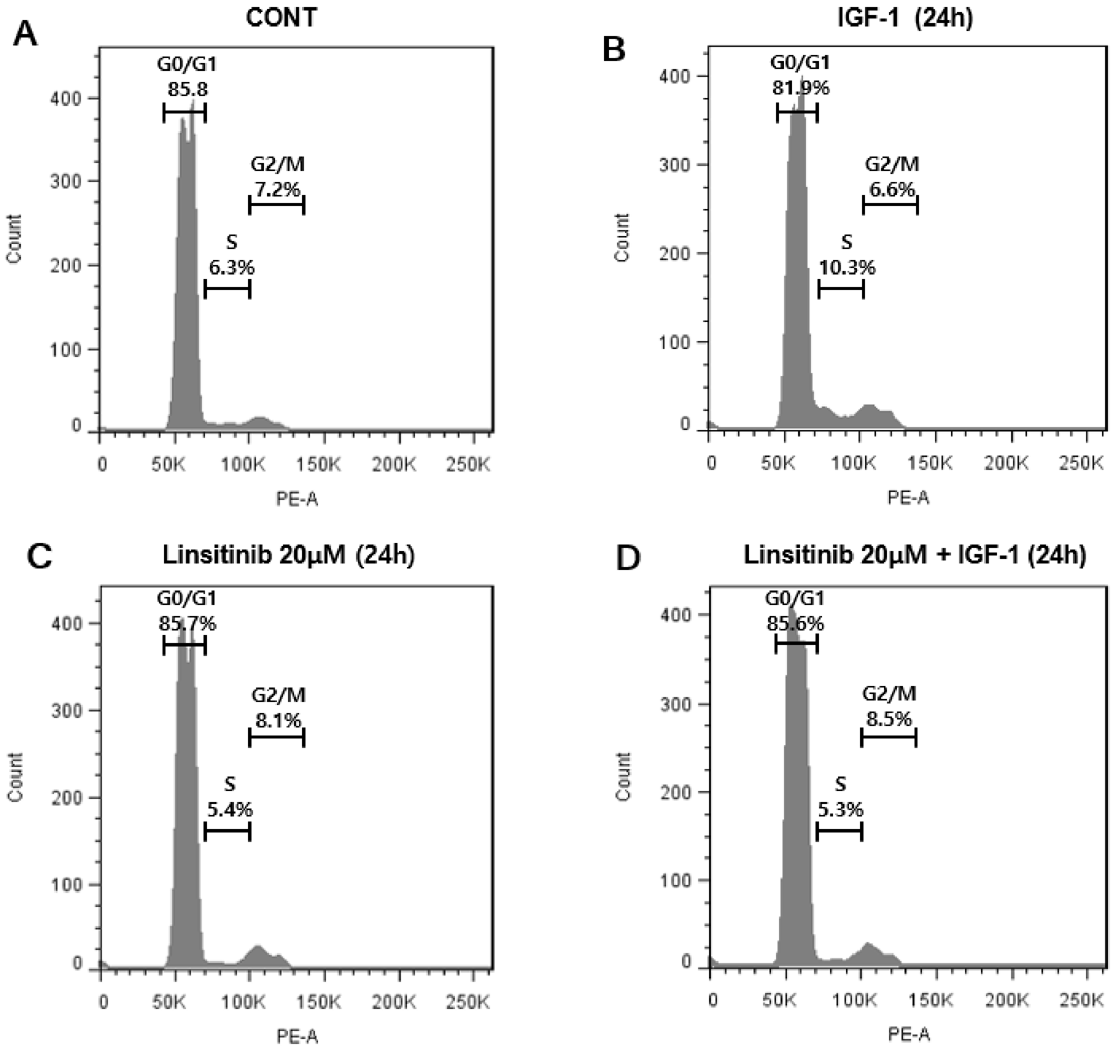


**Figure S1. Pretreatment with linsitinib reduces IGF-1-increased the proportion of S-phase cells in OFs from TAO patients.** Representative histogram of the gated cells in the G0/G1, S, and G2/M phases of linsitinib-pretreated or/and IGF-1-treated OFs from TAO patients. OFs from TAO patients were plated at a concentration of 2 x $10^5$ cells/well in a 6-well plate. After 24 h, the cells were pre-treated with linsitinib at the indicated concentrations for 2 h, followed by treatment with 50 ng/ml IGF-1 for 24 h. After 24 h, cells were collected and stained with propidium iodide (PI) and their DNA contents were analyzed by flow cytometry.
